# Raman Spectroscopy in Skeletal Tissue Disorders and Tissue Engineering: Present and Prospective

**DOI:** 10.1089/ten.teb.2021.0139

**Published:** 2022-10-11

**Authors:** Marco Fosca, Valentina Basoli, Elena Della Bella, Fabrizio Russo, Gianluca Vadalà, Mauro Alini, Julietta V. Rau, Sophie Verrier

**Affiliations:** ^1^ Istituto di Struttura della Materia, Consiglio Nazionale delle Ricerche (ISM-CNR), Via del Fosso del Cavaliere, Rome, Italy.; ^2^ AO Research Institute Davos, Davos, Switzerland.; ^3^ Laboratory for Regenerative Orthopaedics, Department of Orthopaedic and Trauma Surgery, Campus Bio-Medico University of Rome, Via Alvaro del Portillo, Rome, Italy.; ^4^ Department of Analytical, Physical and Colloid Chemistry, Institute of Pharmacy, I.M. Sechenov First Moscow State Medical University, Moscow, Russian Federation.

**Keywords:** Raman spectroscopy, tissue quality control, tissue engineering, bone, cartilage, ligament, tendon

## Abstract

Musculoskeletal disorders are the most common reason of chronic pain and disability, representing an enormous socioeconomic burden worldwide. In this review, new biomedical application fields for Raman spectroscopy (RS) technique related to skeletal tissues are discussed, showing that it can provide a comprehensive profile of tissue composition *in situ*, in a rapid, label-free, and nondestructive manner. RS can be used as a tool to study tissue alterations associated to aging, pathologies, and disease treatments. The main advantage with respect to currently applied methods in clinics is its ability to provide specific information on molecular composition, which goes beyond other diagnostic tools. Being compatible with water, RS can be performed without pretreatment on unfixed, hydrated tissue samples, without any labeling and chemical fixation used in histochemical methods. This review first provides the description of the basic principles of RS as a biotechnology tool and is introduced into the field of currently available RS-based techniques, developed to enhance Raman signals. The main spectral processing, statistical tools, fingerprint identification, and available databases are mentioned. The recent literature has been analyzed for such applications of RS as tendon and ligaments, cartilage, bone, and tissue engineered constructs for regenerative medicine. Several cases of proof-of-concept preclinical studies have been described. Finally, advantages, limitations, future perspectives, and challenges for the translation of RS into clinical practice have been also discussed.

**Impact statement:**

Raman spectroscopy (RS) is a powerful noninvasive tool giving access to molecular vibrations and characteristics of samples in a wavelength window of 600 to 3200 cm^−1^, thus giving access to a molecular fingerprint of biological samples in a nondestructive way. RS could not only be used in clinical diagnostics, but also be used for quality control of tissues and tissue-engineered constructs, reducing number of samples, time, and the variety of analysis required in the quality control chain before implantation.

## Introduction

Musculoskeletal disorders are the most common cause of severe chronic pain and reduced quality of life, representing an enormous socioeconomic concern. Approximately 1.71 billion people globally are affected by functioning limitations and disability.^1^ Musculoskeletal conditions can either arise suddenly, such as fractures, sprains and strains, or develop from chronic conditions. They affect different tissues of the locomotor system such as bone, cartilage, tendons, and muscles. Each of these tissues presents a highly hierarchical organization ranging from macroscale (fibrils, osteons, etc.) to microscale (proteins) and to nanoscale (molecular composition) structures.

In case of disease or injury, current tissue diagnostics and monitoring techniques are mainly based on macroscopic evaluation methods, such as X-ray, ultrasound, computed tomography, dual-energy X-ray absorptiometry (DXA), and magnetic resonance imaging (MRI). If necessary, those techniques are combined with histopathological evaluations and marker identification, or/and metabolic activity assays (e.g., bone turnover related proteins in body fluids). If those methods are critical for diagnostics, they provide limited information regarding the molecular status of tissues and their lesions that are often asymptomatic at early stages.

Therefore, other techniques that are able to increase the reliability of diagnosis and to help through the clinical decision-making process are required. Raman spectroscopy (RS) is very suitable for this goal since it can provide a comprehensive profile of the tissue composition *in situ*, in a rapid, label-free, and nondestructive manner.^2^ Indeed, this technique produces a molecular fingerprint of the constituents of a tissue, based on inelastic light scattering.^3^ Being compatible with water, RS can be performed on unfixed, hydrated tissue samples, without any manipulations, such as molecular labeling or chemical treatments, which represent potential sources of artifacts.

RS is, thus, a promising *in vivo* tool for various biomedical applications, including the musculoskeletal system. In clinical settings, RS was already used to discern aging of tissue and tendon molecular differences,^4^ to evaluate the extracellular matrix (ECM) in osteoporotic and osteonecrotic bone^5,6^ and to predict fragility fractures.^7^ Moreover, it has been used to characterize articular cartilage degeneration^8^ and select the optimal treatment strategy during cartilage repair surgery.^9^

The present review is focused on RS principles, highlighting its orthopedic applications. Both basic science and translational studies on musculoskeletal tissues are reviewed. Finally, usefulness, limitations, future perspectives, and challenges for translation of RS into clinical use are also discussed.

## Raman Spectroscopy

### Principle and techniques

RS is an inelastic scattered photon procedure in which one photon is absorbed whereas another one is emitted. Light getting in contact with transparent fluids emits two types of light scattering: one is determined by the normal optical properties of the atoms or molecules, and the other is determined by their fluctuation from normal state.

When light waves interact with a molecule, they modify their vibrational status inducing small changes (shifts) in the scattered light frequency. When the incident light energy is scattered with equal energy, this refers to Rayleigh or elastic scattering. In the case of Raman scattering, the interaction of light with a molecule induces changes in its intrinsic vibrational state (molecular electrons oscillate in response to the light photon excitation). Those changes translate as a small shift in the frequency of the scattered light: either through a loss (Stokes, most common case) or through a gain of the scattered light energy (anti-Stokes). This is referred to as Raman shift or scattering (inelastic scattering).^10^ Raman scattering is, thus, highly specific to molecular bonds and can be used to identify both molecules and their response to their environment.

A conventional RS setup comprises a laser source—typically in the near infrared as it reduces autofluorescence interferences that are typical for biological specimens,^11^ a series of mirrors, and a microscope directing the light toward the sample. After interaction with the sample, only a small portion of the scattered photons have changed their frequency. After the filtration of elastically scattered photons, photons with modified frequency are directed toward a spectrometer linked to a detector system (CCD) from which the data are transferred to a computer.

**FIG. 1.**
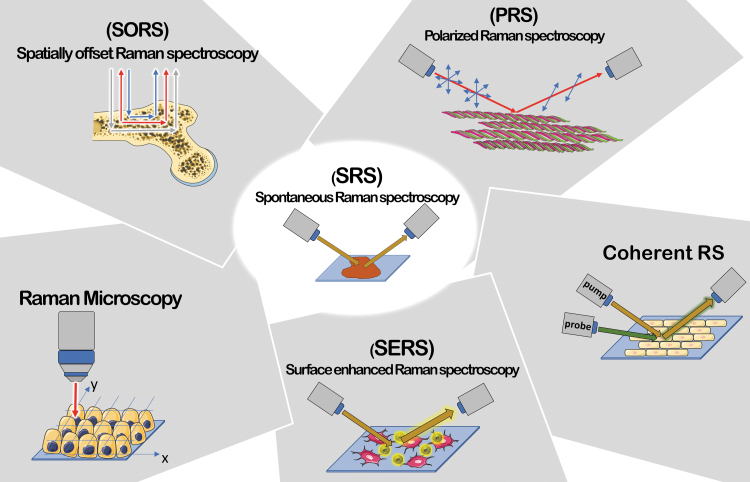
Summary of RS techniques. PRS suitable for anisotropic systems; Raman Microscopy, able to perform correlation between chemical and morphological features; SORS for deep regions signal's collection; coherent RS (SRS and CARS) characterized by an improvement in signal intensity (up to 10^5^ factor) and a faster acquisition; SERS characterized by signal intensity enhancement up to 10^10^ factor due to plasmonic effect between metal particles and sample. CARS, coherent anti-Stokes RS; PRS, polarized RS; RS, Raman spectroscopy; SERS, surface enhanced RS; SORS, spatially offset RS; SRS, stimulated RS. Color images are available online.

However, Raman signal being very weak, many efforts were placed in finding ways to increase it.^12^ As of today, >20 RS techniques have been developed and some of them will be described throughout the present review (Fig. 1).

In spontaneous RS, the Stokes signal is detected under constant radiation. For the RS application *in vivo* for clinical purposes, the light should be delivered directly to the tissue and collected by specifically designed fiber probes^13,14^; however, one of the biggest challenges related to the collection of signal with the use of fiber probes is related to tissue autofluorescence.

The issue mentioned earlier can be overcome by using confocal RS. The combination of a confocal microscope with a Raman spectroscope improves optical depth acquisition. In confocal RS, a spatial filtering of the collected RS light with a pinhole or an optical fiber is applied to block out-of-focus signals. To date, confocal Raman probes are mainly used for *ex vivo* and *in vitro* studies.^15,16^

Compared with RS, spatially offset RS (SORS) collects Raman signal from deeper regions of tissue by spatially offsetting the detection and excitation optical fibers. Collecting the Raman signal at different offsets effectively samples different layers in the tissue. SORS typically uses a probe with an illumination fiber surrounded by detections fibers offset of 1–5 mm,^17,18^ but an offset as high as 16 mm has been used to perform Raman tomographic imaging in bone.^19^

Spectra collected by the SORS technique are usually characterized by convolution of signals originated at different depths of the investigated specimen and can be considered an unwanted consequence of a relative low-depth resolution. Reduced depth resolution relies on two main causes: (1) a relatively limited depth of field in a two-dimensional detector and (2) the geometric aberration induced by axicon lens. These technical limitations can lead to a simple qualitative sampling with a significant cross-talk among different layers.^20^

In the Coherent RS technique, the difference due a vibrational mode frequency generated by two light sources (referred as pump and Stokes beam) results in the acquisition of signal for a specific molecular bond of interest. The coherent addition of the Raman signal from different molecules improves the signal compared with spontaneous Raman, typically by up to ∼10^5^.^21^ Coherent RS techniques include stimulated RS (SRS) and coherent anti-Stokes RS (CARS). Both SRS and CARS can be performed in highly fluorescent media, which is usually a limiting factor for Raman imaging in tissues. In addition, RS delivers the full Raman spectrum of a molecule and, therefore, it is a rather slow procedure; on the other hand, SRS and CARS provide a faster acquisition process, as they only focus on a specific vibrational transition.

Surface enhanced RS (SERS) provides enhanced signals exploiting the effects occurring near a metal surface, including metal nanoparticles, to produce electromagnetic and chemical amplification.^22^ Limitations of SERS for biomedical applications depend on the knowledge of sensitive disease biomarkers and the availability of corresponding targeting molecules, as well as potential toxicity and the need for regulatory approval of the contrast agent.

Polarized RS (PRS) provides information regarding both chemical composition and anisotropic response of highly oriented systems, such as the amide I band of collagen and alpha helical structures with respect to the polarization angle of the incident laser light.

### Spectral analysis

#### Spectral pre-processing

Due to the molecular complexity of biological samples, pre-processing steps of RS spectra are usually required to reduce noise, remove background, and normalize them to enable comparisons between data sets and gain diagnostic information. Autofluorescence is an intrinsic characteristic of biological specimens in optical spectroscopy. Autofluorescence is a consequence of irradiation of UV/Vis light of a specific wavelength on biological specimens. Endogenous fluorophores contained in both cells and extracellular matrices are primarily responsible for fluorescence emission. They are constituted by specific biochemical compounds contained in cells, namely amino acids, lipo-pigments along with pyridinic Nicotinamide adenine dinucleotide phosphate and flavin coenzymes and in ECM contained compound, such as collagen and elastin.^11^

Therefore, Raman spectra quality is affected by stochastic noise.^23^ Specific algorithms performing a baseline correction can help to subtract inelastic scatter fluorescence contribution.^24^ Fourier filtering and polynomial curve fitting are also commonly used for baseline correction.^24,25^ Morris *et al.*^26^ showed that long-lived fluorescence signal could be prevented by applying time gating steps to the spectra acquisition procedure.

#### Spectral analysis and fingerprint identification

The multivariate analysis (supervised and nonsupervised) is one of the most frequently used inquiry tools. Principal component analysis (PCA) represents the most common unsupervised method used for feature extraction.^27–30^ The number of dependent variables can be reduced to principal components (PCs) that are orthogonal and independent by using PCA. Supervised methods can provide a label for the generated classes. Afterward, the classes can be discriminated by a validation method. There are several alternatives for selection and extraction used in RS, including Fisher-based or correlation feature selection, multifactor dimensionality reduction, and non-negative matrix factorization.^31–34^

#### Raman database

To date, free Raman spectra databases are only available for minerals, inorganic materials, or simple organic molecules. For example, the Bio-Rad's SpectraBase (https://spectrabase.com) has more than 24,000 spectra available, mostly of basic organic compounds.

Unfortunately, comprehensive databases of Raman spectra of biological compounds and tissues are still not available due to their complexity and the necessity of standardization of Raman data. Nevertheless, some reviews reported an extensive collection of the most relevant Raman bands that can be found in a Raman tissue investigation. A review by Talari *et al.*^35^ represents today the most complete list of assigned Raman peaks from biological specimens, reporting >1000 assigned bands extrapolated from Raman spectra from both healthy and pathological tissues. Other reviews are also helpful to assess comparison and attribution of the spectra from biological samples,^36^ including lipids^37^ and carbohydrates.^38^

## Raman Studies of Skeletal Tissues

Since the Raman spectrum of H_2_O is very weak and, therefore, does not interact with other molecular signals, RS has become a powerful tool for the analysis of biological samples.^12,39^ Pioneering studies collected Raman spectra of proline oligomers and Poly-l-proline,^40^ amino acids and Poly-l-hydroxyproline,^41^ and of proteins and lysosomes in animal tissues *ex vivo*.^42^ The first Raman report of bovine Achilles tendon showed the comparison in laser excitation scattering among gelatine, skin collagen (solid and solution state), and constituent amino acids.^43^ A few years later, RS was used for cells,^44^ to discriminate live and dead cells,^45–47^ and cell cycle or cell differentiation stages.^45,48–51^

Over the past years, many studies were done on musculoskeletal tissues by using various types of RS. Raman peaks characteristic for various musculoskeletal disorders, their biochemical assignment and relative literature references are reported in Table 1.

**Table 1.** Outline of Various Raman Spectroscopy-Derived Techniques Presented in This Work

Raman technique	Raman shift position (cm^−1^)	Assignment	Skeletal disorder	References
RS	800–950	Tyrosine, proline, and hydroxyproline	Tendinitis, tendon injuries	^70,71^
	1006	Phenylalanine/tryptophan		
	1250–1350	amide III and CH modes		
	1454, 1670	CH_3_/CH_2_ bending and amide I		
	962	Hydroxyapatite	Osteoarthritis	^81^
	1, 1280	Amide III		
	1070	Carbonate		
	1220–1360	Amide III		^124^
	1450, 2840–2986	C-H bond		
	1600–1720	Amide I		
	1500–1700	CN CC, C—N, amide I vibrational bands of DNA, RNA, phenylalanine, tyrosine,	Degenerative lesions of the supraspinatus rotator cuff tear	^72^
	588, 628, 686, and 1503	Monosodium urate	Gout	^89^
	1070	Carbonate	Osteogenesis imperfecta	^105^
	960	Hydroxyapatite		
	1675	Amide I		
SORS	962	Hydroxyapatite	Osteoporosis	^95^
	1070	Carbonate		
	1250	Amide III		
	1450	CH_2_ wag		
	1665	Amide I		
	960	Hydroxyapatite	Osteogenesis imperfect	^104^
	1450	CH_2_ wag		
	1650	Amide I		
	961	Phosphate	Mineralization level (due to general disorder)	^101^
	1071	Carbonate substitution		
	1450	CH_2_ deformation band		
PRS	1250	Amide III	tendon stress strain test	^58^
	1450	C-H bending		
	1665	C=O stretching of the protein backbone		
	937	Collagen backbone	Tendon ageing	^69^
	960	Hydroxyapatite		
	1665	Amide I secondary protein structures		
	853, 934	Proline		^4^
	904	Collagen backbone		
	990, 1003	Phenylalanine		
	1100	Ring-associated and carbohydrate bands		
	1400–1500	CH_2_ bending		
	430, 590, 960	Phosphate	Bone brittleness	^106^
	857, 878	Proline, hydroxyproline		
	1074	Carbonate		
	1248	Amide III		
	1668	Amide I		
micro-Raman	1200–1300	Amide III	Periodontal ligament changes during orthodontic treatment	^73,74^
	1620–1680	Amide I		
	2930	CH_3_ modes		
	960	Hydroxyapatite	Masseter entheses of mandibular reconstruction	^75^
	2940, 1003	Type I collagen		
	784	DNA/RNA	Biochemical abnormalities of anterior cruciate ligament	^76^
	1002, 1030	Phenylalanine		
	
	1101	Isomer conformation in lipids		
	1516	C = C bond stretching mode (carotenoids)		
	1749	Phospholipids		
	962	Hydroxyapatite	Osteoarthritis	^82,86^
	1003	Phenylalanine		
	1064	Proteoglycan		
	1070	Carbonate		
	1280	Amide III		
CARS	960	Hydroxyapatite	Osteoarthritis	^88^

The most relevant Raman bands and their assignments related to specific musculoskeletal disorders corresponding literature references are reported.

CARS, coherent anti-Stokes RS; PRS, polarized RS; RS, Raman spectroscopy; SORS, spatially offset RS.

### Tendon and ligaments

RS was long used to understand the structure of tendon and ligament proteins, such as collagen^43,52,53^ and elastin.^43,54,55^ More recently, studies focused on the analysis of collagen anisotropy and orientation in tendon by using PRS.^56,57^

A dynamical insight into tendon molecular hierarchy adaptations to mechanical stress was provided by studying tendon collagen under strain. For this, a multiscale approach was used, including Raman analysis of an *in situ* loading test. Masic *et al.*^58^ investigated a cross-sectional area of rat tail tendon (RTT) by combining confocal Raman microscopy and an *in situ* stress–strain test.^56^ They also investigated the role of water in collagen structure and mechanical behavior with a combination of RS with X-ray diffraction, revealing tensile stress generated by conformation changes with water removal.^59^ Using Fourier transform infrared and confocal Raman microscopy, the adjustment of the collagen structure to tissue hydration was investigated.^60^

In another study, spectra were obtained after RTT extension.^61^ Spectral analysis provided features such as Raman shift position and Full Width Half Maximum of bands. When plotted as a function of the applied strain, they showed that the 822 cm^−1^ band, representative of collagen backbone C–C, and the 879 cm^−1^ band, arising from carbonyl groups located at the side chain, respectively, decreased and increased with the applied strain. Collagen type I structure was also studied under hydrostatic pressure.^62^

The study of tendon enthesis by RS helped getting more insight on its development and structure.^63^ Traditionally, it was believed that the tendon enthesis was divided into distinct regions, whereas mechanical studies suggested a gradual transition of tissue structure and functional properties. This latter tendon enthesis model was confirmed by Raman-based investigations, demonstrating that it is structured with a gradient of collagen and mineral components and crystallinity.^64–66^

Raman spectral mapping of bone insertion of the anterior cruciate ligaments (ACL) and of rotator cuff tendon (RCT) revealed a similar composition for the two structures, but with a different gradient of mineral content, which was higher for RCT.^67^ These results provide the base for prosthetic engineers to accurately design specific biomaterials for the synthetic ligament. Likewise, the study by Marinovic *et al.* analyzed the role of bone sialoprotein in the structure of the tendon enthesis,^68^ revealing a regulatory role in the zone traditionally associated to the calcified fibrocartilage.

RS can be used as a tool to study tissue alterations associated to aging, pathologies, and disease treatments.^4,69^ RTTs from adult and aged rats were studied by PRS and showed an increase in the anisotropy degree of Raman bands in tendons from old rats; therefore, a higher alignment of the collagen fibers can be associated to aged rat tissue.^69^ In equine tendons, age-related changes in post-translational glycation of collagen were detected and likely correlated to a decline in function.^4^

RS application has been described useful in the studies regarding experimental tendinitis,^70^ tendon injuries,^71^ or degenerative lesions of the supraspinatus RCT.^72^ In the combined magnetic field, some studies dealt with periodontal ligament changes during orthodontic treatments^73,74^ or in mandibular reconstruction.^75^ Moreover, RS was revealed to be useful in supporting the diagnostics of biochemical abnormalities of the ACL.^76^

**FIG. 2.**
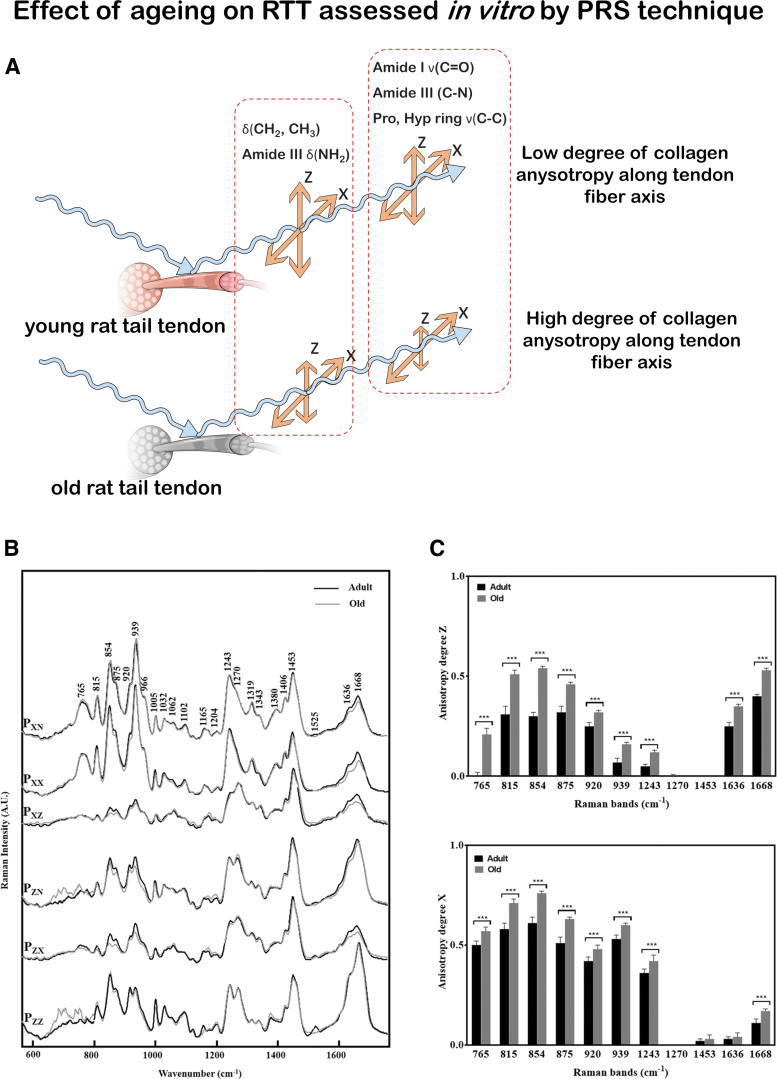
RS for the analysis of tendon aging. **(A)** Schematic representation of the effect of aging on RTT by PRS. **(B)** Mean conventional and PRS spectra measured on adult (*black line*) and old (*gray line*) RTT with the same experimental conditions and compared according to the age. **(C)** polarization anisotropy (Az) and (Ax) calculated on specific collagen bands from adult (*black bar*) and old (*gray bar*) RTT. Intensity ratio of different Raman bands can provide anisotropy degree of collagen backbone along the tendon fiber axis. A higher degree of anisotropy characterized old-aged tendon in comparison to the young one. **(B, C)** are adapted with permission from Van Gulick *et al.* (doi.org/10.1038/s41598-019-43636-2).^69^ RTT, rat tail tendon. Color images are available online.

Schematic representation of *in vitro* musculoskeletal assessment conducted by PRS technique (as described in Ref.^69^) is reported in Fig. 2. Figure 2A shows a scheme of the PRS investigation performed on both old and adult RTT fibers to assess the effect of aging on the tissue. In Figure 2B, it is shown how collected spectra have been compared by age for each polarization direction. Figure 2C shows the anisotropy degree (Az) and (Ax) of different Raman Bands compared according to RTT age.

### Cartilage and subchondral bone

Cartilage is principally composed of chondrocytes-produced ECM, primarily consisting of water, type II collagen fibrils, proteoglycans, and hyaluronic acid. The cartilage is organized in three zones with different spatial organization, collagen and proteoglycan content, and orientation, resulting in different mechanical and biological properties. Calcification of cartilage occurs to fix the collagen fibrils onto the adjacent bone (subchondral bone). The subchondral bone has the same composition as the cortical bone (see next paragraph).^77^

Raman spectra of healthy articular cartilage share several features with tendon and ligament spectra. Collagens represent the main component of both cartilaginous tissue and tendon. However, a band at ∼1063 cm^−1^, arising from the sulfated glycosaminoglycans (GAG), unique to cartilage, distinguishes its spectrum from that of other soft tissues and bone.^78,79^ These observations using Raman technique were recently confirmed by fiber-based fluorescence lifetime technology, allowing for real-time study of the structural, compositional, and molecular contrast of cartilage.^80^

Osteoarthritis (OA) is a degenerative condition affecting articular cartilage and the whole joint, including the synovium and the osteochondral bone. In a preclinical rat model^81^ and in clinical setups,^82^ RS could distinguish between different OA severity grades. An RS analysis of synovial fluid from 40 patients with knee OA correlated with the pathology severity scores obtained using Kellgren/Lawrence score, which is based on X-rays analysis.^83^ Cartilage calcification and water contents phenomenon also correlate with cartilage degeneration independently of age, and this can be used as a potential marker for early detection of joint degeneration.^84^

Changes in the water content in cartilage tissue comprise an early diagnostic of OA, and they can help in the classification among different grades.^80,82,85^ Among pathology spectra, the quality of cartilage was identified following distinct chemical fingerprints for the nonochronotic, compared with ochronotic cartilage.^86^ Moreover, it has been demonstrated that the progression of OA can be followed by evaluating chondrocyte differences using the amide I (1612–1696 cm^−1^), amide III (1229–1300 cm^−1^), and phenylalanine (1001–1007 cm^−1^) bands; in addition, OA progression is also correlated with a decrease of the nucleic acid content (780–794 cm^−1^ band).^87^

**FIG. 3.**
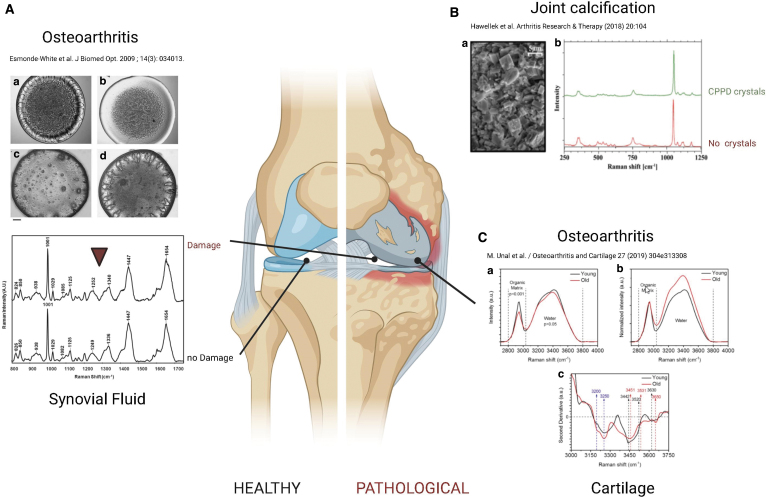
RS for the analysis of healthy and diseased joints. **(A)** The biochemical composition of the synovial fluid from healthy subjects and OA patients was analyzed by RS, showing the differences in protein secondary structures and content from OA patients. Figures are reproduced with permission from Esmonde-White *et al.* (doi: 10.1117/1.3130338).^83^ **(B)** Differences in Raman Spectra can be found in diseased acetabular joint calcification. Figure reproduced with permission from Hawellek *et al.* (doi: 10.1186/s13075-018-1595-y).^84^ **(C)** Variations in the water content in cartilage specimen showed altered spectra in young and old patients by RS. Figure reproduced with permission from Unal *et al.* (doi:10.1016/j.joca.2018.10.003).^85^ Figure created with Biorender.com. OA, osteoarthritis. Color images are available online.

The biochemical composition of the ECM in the spongy and subchondral bone in patients with femoral neck fractures without osteopathic arthritis was examined,^5^ demonstrating that Raman microspectroscopy can show alterations for osteopathic arthritis, characterized by relevant changes in hydroxyapatite (HA)-to-collagen ratio, carbonate apatite-to-HA ratio and disorganization in collagen secondary structure via amide III. SRS and CARS were used to analyze tissue microstructure in the region between calcified cartilage and subchondral bone, showing that cartilage microstructure differs between regions subjected to different loadings in the deep areas, which might be important for mechanical interactions of cartilage and subchondral bone (Fig. 3).^88^

RS can be also used for the diagnosis of other joint pathologies, such as gout, characterized by the deposition of monosodium urate in the joints and other organs. Through RS, it is possible to assess non-invasively (detecting through the skin) the presence of crystals situated around the first metatarsophalangeal joint.^83,89^

### Bone

Bone is a multiscale mineralized tissue consisting of cortical bone, composed of highly structured and oriented osteon, and trabecular bone composed of interconnected trabecular rods.^90,91^

At the microscopic level, bone is a composite material containing an inorganic (mainly apatite crystals, calcium salts, and water) and an organic phase comprising different types of cells from the mesenchymal and the hematopoietic lineages.^92^ Giving access to the organic and inorganic components of bone, their composition, orientation, crystallinity, and degree of crosslink, RS also gives access to bone quality and indirectly mechanical properties on aging, diseases, or injuries and recoveries in a non-or minimal invasive way.^93–95^

At the ultrastructural level, bone matrix is composed of type I collagen organized in mineralized collagen fibrils, which are further assembled in large collagen fibers.^96–98^ The orientation and alignment of those collagen fibers together with the amount and quality of the inorganic phases contributes to the macrostructural organization of bone tissue and, thus, to its mechanical properties. Giving access to information at the molecular level, RS enables the quantification of bone quality related parameters, such as minerals, matrix and their relative ratio, crystallinity, carbonate substitution, as well as collagen fiber primary and secondary structures.^97,99,100^

Photon migration into the bone and Raman signals arising from different depths of selected bones with various mineralization levels were analyzed by SORS.^101^ In other work, Raman signals were retrieved in depths up to about 5 mm, with the penetration depth depending on the degree of bone mineralization. Besides, PRS gave access to the collagen fibril content, orientation, and crystallization status of cortical bone, whereas spongy bone was characterized up to the microstructural level, showing that the ν_1_PO_4_^3^−/amide I ratio, susceptible to polarization effect, brings information on collagen fiber orientation.^98,99,102^

Giving a quick access to bone mineral and organic components and, therefore, to biomechanical properties of bone,^93^ RS also became a promising method for the detection of other bone- and mineral-related diseases, such as osteoporosis^95,103^ and osteogenesis imperfecta.^104,105^

For the study of osteoporosis, Shu *et al.*^95^ proposed the use of SORS in a transcutaneous approach to predict bone quality in a mouse model. Despite the overlay tissue interferences (mainly due to type I collagen), they demonstrated a strong correlation between bone quality metrics measured by Raman and detected by classical micro-CT, DXA, and mechanical testing methods. Similarly, another study showed a strong correlation between bone mineral contents and fracture risks, but also and more importantly the involvement of the bone collagen components, essentially the amide I region of the Raman spectra.^103^

The SORS can detect subcortical tissue in the long bones of mice and rabbits, and it is sensitive to biochemical changes in a mouse model with imperfect osteogenesis.^104^ On the other hand, PRS revealed a correlation between mineral and collagen orientations and the mechanical phenotype of bones, whereas differences in composition alone failed to fully explain the differences in toughness between different disease genotypes.^106^

Fracture repair is a complex process involving many factors and steps aiming at restoring the biological and mechanical function of bone.^107^ If bone shows a high regenerative and healing capacity, fractures still fail to heal, leading to delayed union or nonunions, and large bone defects. A better understanding of the healing course at all scales will enable the understanding of the possible causes of failures, crucial for the development of appropriate therapies or replacement strategies.^108^

Many *in vitro* studies have been performed at the cellular level. In a subcritical rat calvaria defect, Ahmed *et al.* compared bone composition changes after a 7 or 14 days healing period, and with intact bone.^109^ RS data—that is, the mineral matrix ratio (ʋ1 PO_4_^−3^/amide III, ʋ1 PO_4_^−3^/CH_2_ wag), the carbonate/phosphate ratio, and the crystallinity corroborated with the *in vivo* callus formation stage. Recent studies investigated the application of the SORS to monitor bone healing *in vivo*.^101,110,111^

**FIG. 4.**
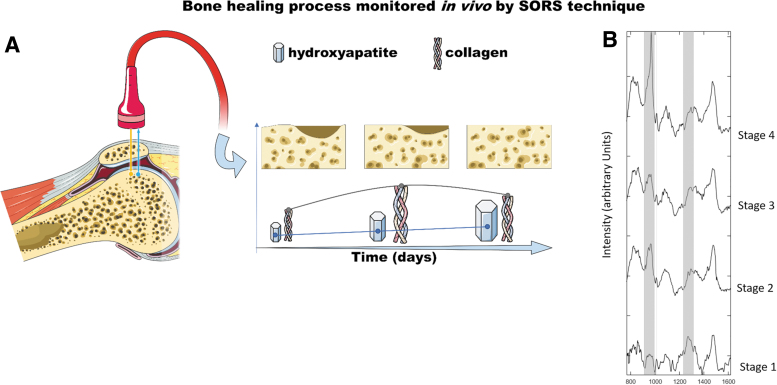
*In vivo* monitoring of bone healing process by SORS. **(A)** SORS can provide Raman signal arising from a defined depth, allowing to collect biochemical data from inner layers of living tissue. Intensity ratio of collagen (amide I, III) and HA (phosphate) Raman bands furnish indication of bone healing status. **(B)** SORS spectra (after substraction of baseline) of collagen:HA phantoms inserted in rat skulls. Different stages of bone regeneration are characterized by specific collagen:HA ratio. Figure is reproduced with permission from Dooley *et al.* (doi: 0.1002/jbio.202000190).^110^ HA, hydroxyapatite. Color images are available online.

A schematic representation of an *in vivo* clinical study carried out using the SORS technique (as described in Dooley *et al.*^110^) is reported in Figure 4. Part (A) is a representation of the bone healing process monitored *in vivo* by SORS. Intensity ratio of collagen and HA Raman bands can provide reliable information on the healing process as a daily trend. Part (B) reports the comparison of SORS spectra collected at different stages, of the healing process, upon collagen:HA phantoms inserted in rat skulls. Different stages of bone regeneration are characterized by specific collagen:HA ratio.

### Tissue engineered constructs for skeletal tissue repair

In case of natural tissue healing failure, autografts, allografts, or tissue engineered (TE) constructs are foreseen. In the three earlier mentioned musculoskeletal tissues (tendons, cartilage/subchondral bone, and bone), the lifetime and success of the grafted material depend on its mechanical properties and rapid integration in native tissue. Typically, TE constructs comprise a scaffold showing specific requirements of the host tissue associated with tissue-specific cells or stem cells aiming at restoring structural, mechanical, and biological functionality of the injured tissue on implantation. A major challenge in this field is the possibility to recapitulate the biological, mechanical, and structural composition of native tissue in an implantable construct. A successful clinical translation of TE constructs requires robust quality control before implantation and Good Laboratory Practices certified fabrication procedures.

#### Cells

Autologous mesenchymal stromal cells (MSC) from bone marrow or adipose tissues are commonly used in TE constructs for bone, cartilage, or tendon repair. Being a heterogenous population, MSC differentiation and cell growth capacity differ among donors.^112^ This lack of reproducibility, together with the difficulty to separate cell sub-populations, constitutes the main drawback to MSC clinical use. To date, cell characterization and subpopulation identification can only be assessed via methods, such as flow cytometry, gene expression analysis, and immunocytochemistry; most of these methods involve the destruction of the original sample and are time consuming. As mentioned earlier in this review, RS showed the possibility to distinguish between live and dead cells,^46,47^ as well as cells in different phases of their cycle.^45,48,49,51^

**FIG. 5.**
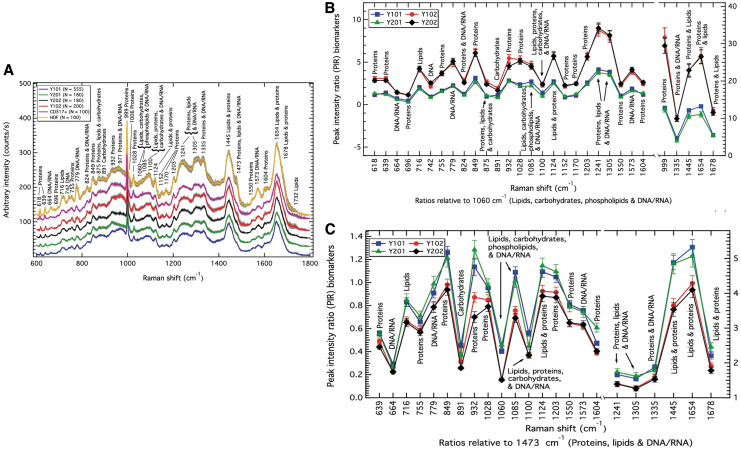
Mesenchymal stem cells characterization **(A)** Average Raman spectra measured from four MSC lines originated from a common MSC pool. MSC positive control: CD317+MSC. MSC negative control: HDF. PIR analysis of the spectra showed the discrepancy between differentiation-competent cells sub-population, and differentiation incompetent cells sub-population. Raman spectra showed discrepancies between the groups at the DNA/RNA, carbohydrates, lipids, and proteins levels (1060 cm^−1^ band) **(B)**, and the protein, lipids, and DNA/RNA-related band (1473 cm^−1^) **(C)**. Figures are reproduced with permission from Rocha *et al.* (doi:10.1038/s41598-021-81991-1).^113^ MSC, mesenchymal stromal cells; PIR, peak-intensity-ratio. Color images are available online.

In a recent publication, Rocha *et al.*^113^ proposed RS to quantitatively characterize clonal MSC, and its use as a label-free biomolecular characterization tool of MSC and sub-populations in a mixed population. In this study, the authors created four immortalized cell lines out of four single-cell-derived colonies from a heterogenous cell population. Of those, two cell lines showed osteogenic, chondrogenic, and adipogenic differentiation capacity (Y101, Y201), whereas the two other groups did not (Y102, Y202). Raman spectra analysis performed on the nuclear region of those cell subpopulations clearly showed signal discrepancies between the competent and non-competent cell population, as depicted by their overall Raman spectra (Fig. 5A).

Comparing the 4 MSC sub-populations with CD317-positive MSC (MSC-positive control), or with dermal fibroblasts (MSC negative control, HDF), key differences mainly occurred at the protein (932 cm^−1^ band) and proteins DNA/RNA bands (971 cm^−1^) but also in the region of the lipids, phospholipids, carbohydrates, and DNA/RNA region of the spectrum (1060 and 1085 cm^−1^). Comparing the four MSC sub-populations (tri-lineage differentiation competent, or tri-lineage differentiation incompetent), main differences were observed in the protein region (932 cm^−1^), and the protein and DNA/RNA region (971 cm^−1^).

The authors further analyzed the Raman spectra of the four different MSC lines comparing all peaks' intensity between each other (peak-intensity-ratio [PIR] analysis) and could discriminate between differentiation-competent cells and differentiation-incompetent cells. The PIR panels that predominantly distinguish between those two groups of biological response were found in the DNA/RNA, carbohydrates, lipids, and proteins (1060 cm^−1^) (Fig. 5B), and the protein, lipids, and DNA/RNA region (1473 cm^−1^) (Fig. 5C).

#### Matrix

Cell differentiation phases can be monitored by analyzing matrix deposition and mineralization in contact with biomaterial surfaces.^49,114–117^ ECM is a fundamental component of all living tissues; it is synthetized by tissue-specific cells and provides mechanical, structural, and biochemical support. Parallel to phenotype-specific gene expression, ECM deposition is a key indicator of cell differentiation and a crucial factor in tissue integrity, repair, and the development of TE constructs. However, as for cell characterization, methods to analyze matrix deposition are mainly invasive, long-term, and time-consuming. Thus, the strength of using RS in TE relies on the possibility to conduct sample analysis directly at the ECM biomolecules level in a noninvasive way.

With the aim of monitoring changes in the properties of collagenous tissues, Shaik *et al.*^118^ used several non-destructive techniques, including RS, to monitor collagen digestion phases over time at the molecular level. Raman spectra intensity in the region of the proline, hydroxyproline, C-Cα stretch, and amide I region decreased over time. They also showed that with the longer digestion time (32 h) type I collagen characteristic peaks (proline, amide III, and C-Cα) disappeared, demonstrating the usability of RS in monitoring extra-cellular matrix proteins over time in a nondestructive manner.

**FIG. 6.**
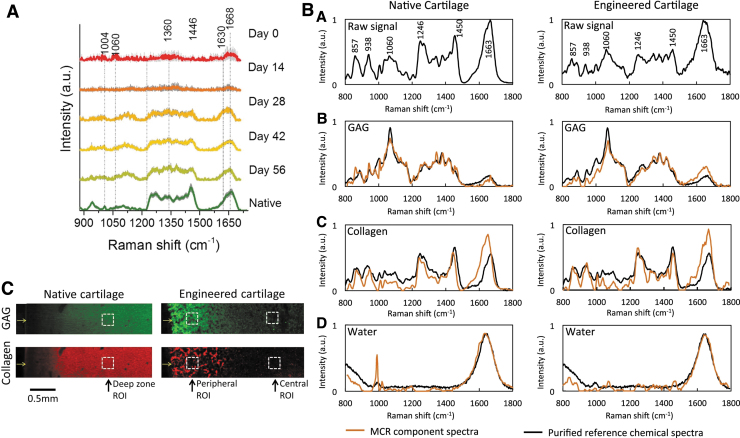
Quantitative and qualitative evaluation of TE constructs. **(A)** Mean Raman spectra of TE cartilage construct cultures over time and native cartilage. Figure is reproduced with permission from Bergholt *et al.* (doi:10.1016/j.biomaterials.2017.06.015).^119^ **(B)** *Upper row*: spectra of native cartilage and engineered cartilage. *Second, third, and fourth rows*: protein-specific spectra compared with correspondent purified proteins. **(C)** Raman semi-quantitative imaging of GAG and or collagen in native or TE cartilage. **(B, C)** are reproduced with permission from Albro *et al.* (doi:10.1038/s41536-018-0042-7).^120^ GAG, glycosaminoglycans; TE, tissue engineered. Color images are available online.

The use of diffuse near-infrared fiber-optic Raman spectra showed its efficacy in the analysis of TE cartilage constructs. Using this noninvasive approach, Bergholt *et al.* quantified the ECM components and showed a high correlation between RS output and classical biochemical assays for the measurement of collagen and GAGs (Fig. 6A).^119^ In a study published in 2018, Albro *et al.*^120^ investigated the usability of RS in the characterization TE cartilage constructs compared with native cartilage tissue. As shown in Figure 6B, Raman spectra obtained from TE constructs indicated similar protein contents to native cartilage, though Raman images calculated from the collagen and GAG Raman spectra, which depicted local heterogeneity between native and TE cartilage (Fig. 6C).

#### Cell-matrix interactions

Early cell-material interactions are determinant for the further evolution of TE constructs. After sedimentation on a biomaterial surface, cells are sensing their environment in terms of chemistry, surface topography and stiffness, as well as possible added biological cues. On adhesion involving specific cell molecules, such as integrins, cells start to produce their own ECM, promoting their growth, differentiation, further matrix deposition, and eventually matrix mineralization, in the case of bone TE. Cell-material interactions are usually studied through metabolic activity assays to assess cell viability and growth or using imaging techniques involving cell fixation and staining with antibodies. Recently, RS has shown the ability in monitoring the mineralization of bone nodules for *in vitro* bone TE applications.

**FIG. 7.**
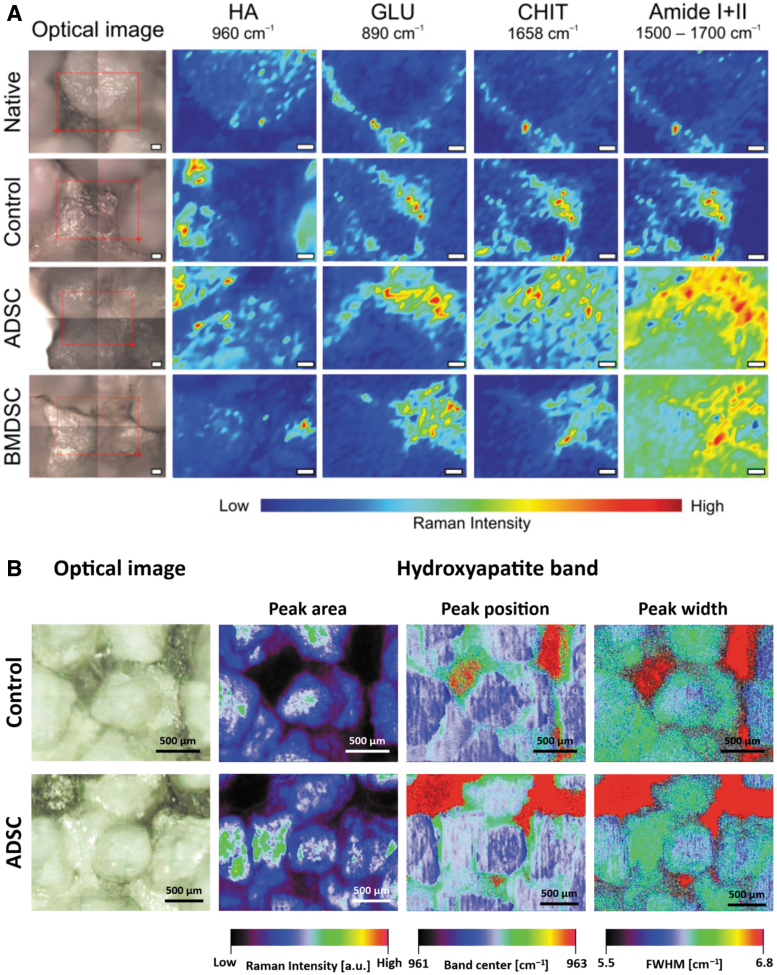
Raman assessment of cell-scaffolds interface. **(A)** Chemical mapping of scaffolds components in the presence or not of MSC. Amide I + II shows the distribution of the ECM at the MSC scaffold interface. **(B)** Raman imaging of HA phosphate deposition on scaffolds seeded or not with adipose-derived MSC. Figures are reproduced with permission from Kalisz *et al.* (doi:10.3390/ijms22020485).^121^ ECM, extracellular matrix. Color images are available online.

Cell differentiation can also be monitored on matrix deposition and mineralization in contact with biomaterial surfaces.^49,114–116^ In a recent study, by combining classical RS spectra analysis, Raman spectral imaging, and mapping, Kalisz *et al.* could have access not only to the biochemical changes at the interface between stem cells and osteo-inductive scaffolds, but also to the three-dimensional deposition of the ECM components and mineralization at the cell-material interface.^121^ In their study, the authors first measured the Raman profiles of the three raw components (Chitosan [CHI], beta 1,3 glucan polysaccharide, and HA) that they used in the fabrication of theirs scaffolds, and they monitored the matrix deposited by MSCs from adipose tissue and bone marrow (Fig. 7). The authors could identify molecular differences, spatial distribution, and relocalization between cell-seeded materials and raw materials (Amide I, II, and raw materials [CHI], beta 1,3 HA) (Fig. 7A). They could also have access to HA deposition and crystallinity along the ECM deposition by MSCs (Fig. 7B).

## Status and Challenges for Clinical Translation

According to the most recent available literature concerning the musculoskeletal applications of RS, there are several proof-of-concepts on preclinical studies, which might be translated into clinics. One of them is regarding the investigation of possible crystals such as monosodium urate for gout, OA or rheumatoid arthritis, and calcium pyrophosphate in pseudogout in joint and synovial fluid using customized RS.^122,123^

RS can be used intraoperatively to improve surgical treatments for joint diseases or injuries.^79,124^ Matsunaga *et al.*^76^ reported the application of RS for the nondestructive diagnosis of molecular tissue degeneration, applied to *ex vivo* human ACL. Encouraging outcomes of this work could promote Raman microscopy as a reliable alternative to the invasive histology or to MRI, which is unable to identify the biochemical components related to degeneration.

A new method to address early diagnosis of OA was based on hydration status of cartilage assessment by RS, showing a nondestructive quantification of various water zones in cartilage, and calculation of up to 82% of the variance found in the permeability and combined modulus of articular cartilage.^85^ Due to its simplicity and feasibility, this methodology can be used clinically during arthroscopy procedures to control cartilage consistency in a noninvasive or minimally invasive way with Raman probe. In addition, the RS was adapted for arthroscopy of joint cadaveric knee tissues by applying a custom-built fiber-optic probe.^79^ Fiber-optic Raman spectra were compared with the reference spectra of cartilage, subchondral and cancellous bone collected by Raman microspectroscopy. This proof-of-concept study provided a basis for further development of arthroscopic Raman optical fiber probes for application in clinics.

During the past few years, many works were published reporting the use of Raman-based tools for *in vivo* and intraoperative conditions.^125,126^ However, for clinical translation, several requirements need to be fulfilled. In a nutshell, the general definition of standards for technical characteristics of Raman apparatus and for operative parameters, and the use of a standard algorithm for real-time data analysis and interpretation are needed. Despite the existing literature showing that RS stands as a reliable and mature tool for clinical diagnosis and intraoperative procedure, to date no specific reports or ISOs for the clinical use of RS-based tools or procedures are available.

These issues triggered a large-scale cross-laboratory study,^127^ involving 35 different spectroscopic devices Europe-wide. This study aimed at defining how different measurement conditions and experimental setups can affect the quality and differences of Raman spectra, focusing on the four most important metrics defining Raman profiles: peak width, peak shift, signal-to-noise ratio, and specific peak ratios. The authors concluded that the standardization of these parameters is a fundamental step for the definition of a common steppingstone to move RS from laboratories toward real-life application, such as RS use as bioanalytical protocol for clinical applications.

## Conclusion

In conclusion, RS represents an important tool and has great clinical potential in the examination of the musculoskeletal system. It provides deeper insights into comprehensive molecular composition of tissues through *in situ*, label-free, and nondestructive measurements, paving a new way in the life sciences. Further, Raman probe microscopy associated with chemometric-generated hyperspectral imaging can provide a real-time, augmented visualization of the region of interest, allowing for fast recognition and distinction of tissues and their biochemical status.
